# Effectiveness of a Mindfulness Program in Reducing Academic Stress among University Students

**DOI:** 10.1192/j.eurpsy.2026.11820

**Published:** 2026-07-31

**Authors:** K. L. Perez Correa, N. Renteria Valencia

**Affiliations:** 1 Magdalena, Institución Universitaria del Caribe, Ciénaga, Colombia; 2 Universidad Técnica Luis Vargas Torres de Esmeraldas, Esmeraldas, Ecuador

## Abstract

**Introduction:**

Academic stress negatively impacts students’ well-being and performance. Mindfulness has shown effectiveness in reducing stress and improving emotional regulation. This study assessed the impact of a Mindfulness program on academic stress among university students in Ecuador.

**Objectives:**

To evaluate the effectiveness of a Mindfulness program in reducing academic stress among students at the School of Social Work, Luis Vargas Torres Technical University (Ecuador).

**Methods:**

A quantitative quasi-experimental study with pre- and post-intervention measures was conducted. The sample consisted of 46 students divided into two groups (experimental and control, n=23 each). The *Academic Stress Inventory (SISCO SV-21)* and the *Academic Stressors Scale (ECEA)* were applied. The intervention included 16 Mindfulness sessions adapted to the university context.

**Results:**

A significant reduction in academic stress levels was observed in the experimental group following the Mindfulness intervention. Statistical analysis using the Student’s *t*-test for related samples showed a highly significant difference between pre- and post-test scores (*p* < 0.0001), indicating a strong effect of the intervention on stress reduction. The mean stress score decreased from 63.52 (SD = 11.59) to 37.71 (SD = 23.40), representing a reduction of 40.7%. In contrast, the control group showed no significant differences between pre- and post-test scores (*p* > 0.05), maintaining relatively stable levels of academic stress throughout the study period. These findings demonstrate that the positive changes were attributable to the Mindfulness program rather than to external or temporal factors.

**Table 1.** Table 1. Long description.

Group	N	Pretest (M ± SD)	Posttest (M ± SD)	p
Experimental	23	63.52 ± 11.59	37.71 ± 23.40	<0.0001
Control	23	61.84 ± 12.07	60.13 ± 11.89	ns

Additionally, qualitative feedback collected at the end of the program revealed improvements in concentration, emotional regulation, and academic motivation among participants, further supporting the quantitative results.

**Conclusions:**

The Mindfulness program proved to be a highly effective strategy for reducing academic stress and promoting psychological well-being among university students. The results confirm that systematic training in attention, breathing, and self-awareness can enhance coping strategies and emotional regulation in demanding academic contexts. The program’s impact suggests its potential inclusion as a permanent component of institutional health promotion policies in higher education. Integrating Mindfulness-based interventions can contribute to reducing burnout, improving academic performance, and fostering resilience and mental health literacy within the student population. Future studies are recommended to explore longitudinal effects and expand the intervention to diverse educational settings.

**Disclosure of Interest:**

None Declared

## Long descriptions

### Table 1. Long description

The table consists of five columns: Group, N, Pretest (M plus or minus S D), Posttest (M plus or minus S D), and p.

* The first row contains data for the Experimental group with an N of 23. The Pretest score is 63.52 plus or minus 11.59. The Posttest score is 37.71 plus or minus 23.40. The p-value is less than 0.0001.

* The second row contains data for the Control group with an N of 23. The Pretest score is 61.84 plus or minus 12.07. The Posttest score is 60.13 plus or minus 11.89. The p-value is listed as n s (not significant).

Navigate back to Table 1..

